# Research on the Prediction Method for Ultimate Bearing Capacity of Circular Concrete-Filled Steel Tubular Columns Based on Random Search-Optimized CatBoost Algorithm

**DOI:** 10.3390/ma19071360

**Published:** 2026-03-30

**Authors:** Zhenyu Wang, Yunqiang Wang, Xiangyu Xu, Zihan Zhang, Yaxing Wei, Dan Luo

**Affiliations:** 1 Institute of Defense Engineering, AMS, PLA, Beijing 100850, China; wangzz1129@163.com (Z.W.);; 2 School of Water Conservancy and Transportation, Zhengzhou University, Zhengzhou 450001, China

**Keywords:** concrete-filled steel tube (CFST), ultimate bearing capacity, machine learning, hyperparameter optimization, interpretability

## Abstract

With the development of various emerging structures, concrete-filled steel tubular (CFST) columns have become critical load-bearing components in key infrastructures such as subways and underground utility tunnels. Accurately predicting their ultimate bearing capacity (*N_u_*) is essential for guaranteeing structural safety. To address the limitations of traditional empirical formulas and code-based calculation approaches, this paper proposes a prediction model for ultimate bearing capacity based on the CatBoost algorithm optimized by Random Search. Furthermore, the marginal contribution of each key feature to the prediction results is measured through interpretability analysis. First, a database containing 438 CFST column ultimate bearing capacity test cases was established, with key parameters such as geometric dimensions and material properties as input variables. Second, the predictive performance of six machine learning algorithms—CatBoost, LightGBM, Random Forest (RF), Gradient Boosting (GB), K-Nearest Neighbors (KNN), and XGBoost—was compared. A five-fold cross-validation integrated with a Random Search strategy was employed for joint hyperparameter optimization. The results show that the optimized CatBoost model significantly outperforms other algorithms and conventional design codes, achieving a coefficient of determination (*R*^2^) as high as 0.99 and a root mean square error (RMSE) of 174.29 kN. Furthermore, the SHAP (Shapley Additive exPlanations) method was used to perform global and local interpretability analyses of the prediction model. This not only quantified the individual contribution and interaction effects of each feature parameter on the bearing capacity but also revealed that geometric parameters are the primary influencing factor. This finding confirms a high degree of consistency between the prediction mechanism of the data-driven model and classical mechanical theories, effectively validating the model’s reliability. This study provides an efficient and reliable tool for the optimal design and rapid evaluation of CFST columns and establishes a new data-driven paradigm for the design and reinforcement of key components in underground structures.

## 1. Introduction

Concrete-filled steel tube (CFST) structures have become a preferred structural form in high-rise buildings and underground space development owing to their exceptional load-bearing capacity, ductility, and cost-effectiveness [1,2,3]. Particularly in underground infrastructure, such as deep excavation support systems and subway station columns, CFSTs significantly enhance the structural capability to withstand high in situ stress environments by leveraging their compact cross-sections and the effective confinement provided by the steel tube to the concrete [4]. Nevertheless, the complex environments and demanding load scenarios typical of underground engineering place stringent requirements on the accuracy and reliability of CFST capacity predictions. Conventional approaches primarily rely on empirical design formulas and finite element modeling (FEM). Empirical formulas are usually calibrated on limited datasets and may be overly conservative or unreliable under complex loading conditions. Conversely, while FEM can achieve high fidelity, it comes at the cost of substantial computational effort, restricting its utility for rapid design iteration. Accordingly, there is a clear need for a prediction method that delivers both high accuracy and high efficiency, which is of significant value for engineering design.

In recent years, the accumulation of experimental data has substantially enriched research on the mechanical behavior of CFST structures. Scholars such as Le et al. [5], Li et al. [6,7], and Wang et al. [8] have systematically investigated the effects of aspect ratio, complex cross-sectional geometries, long-term loading, and high-temperature environments on CFST bearing capacity through extensive experimentation and numerical simulation. These investigations have not only clarified fundamental resistance mechanisms but also established high-quality datasets that underpin subsequent model development. Based on these datasets, data-driven approaches have been increasingly adopted in this field [9,10]. Machine learning (ML), leveraging its robust nonlinear mapping capabilities, has been widely adopted for structural performance prediction [11,12,13,14,15]. For instance, Mohammad et al. [16] compared six algorithms, including SVM and ANN, demonstrating that ML models outperform existing design codes in accuracy. Lou et al. [17] proposed a knowledge-enhanced learning framework to improve prediction precision for eccentrically loaded columns, while Wei et al. [18] derived simplified design formulas based on ANNs. Kazemi et al. [19] developed an ensemble learning framework that combines synthetic data generation with heuristic optimization, achieving precise predictions of the full mechanical curves and axial compressive capacity of concrete-filled timber-steel tube (CTFST) composite members. This work represents the latest advancements in utilizing ensemble learning to handle increasingly complex composite components and to predict the entire mechanical response process. However, significant gaps remain. Existing studies typically rely on comparisons of single or limited algorithms, lacking systematic evaluations that encompass advanced ensemble methods such as CatBoost and LightGBM. Moreover, standardized protocols for hyperparameter optimization are often overlooked. Furthermore, while current research focuses heavily on enhancing prediction accuracy, it frequently neglects model interpretability [20,21,22,23]. Although interpretability tools such as SHAP have been introduced in related fields [24,25], quantitative insights into how critical variables govern CFST capacity through nonlinear and interactive mechanisms remain insufficient, particularly under conditions typical of underground engineering. This inherent “black-box” opacity erodes engineering confidence in model outputs, thereby constraining the practical adoption of ML-based approaches in structural design.

This paper presents a novel framework for predicting the axial compression capacity of CFST that balances computational accuracy with engineering transparency. Utilizing a validated database of 438 specimens, a rigorous benchmarking of six ensemble learning algorithms was performed. The CatBoost model, optimized via random-search hyperparameter tuning and validated through a five-fold cross-validation scheme, demonstrated superior predictive performance. Furthermore, to mitigate the opacity of data-driven approaches, SHapley Additive exPlanations (SHAP) were utilized to interpret the model, explicitly quantifying the nonlinear impacts and synergistic effects of geometric and material variables on axial capacity. By bridging the gap between stochastic modeling and structural mechanics, this framework serves as a vital instrument for the reliability-based design of CFST structures in underground engineering contexts.

## 2. Dataset and Feature Analysis

### 2.1. Database Compilation

A comprehensive database comprising 438 CFST specimens under axial compression was established by compiling 38 independent experimental datasets from Ref. [26] and the original data sources are provided in the Appendix A. Each data point in the repository is characterized by eight parameters. The input features include the external diameter (*D*/mm), wall thickness (*t*/mm), column length (*L*/mm), concrete size effect coefficient ($\gamma_{U}$), yield strength of the steel tube (*f_y_*/MPa), compressive strength of the concrete prism (*f_pr_*/MPa, based on 150 mm × 150 mm × 450 mm dimensions), and the confinement factor (*Φ*). The ultimate load-carrying capacity (*N_u_*) serves as the target output variable.

The dataset was randomly split into a training set and a test set using a 70:30 ratio, yielding 307 and 131 samples, respectively. The training set was used for model fitting and hyperparameter optimization, while the test set was reserved for evaluating the generalization performance of the developed models.

### 2.2. Statistical Description of the Database

**Figure 1:**
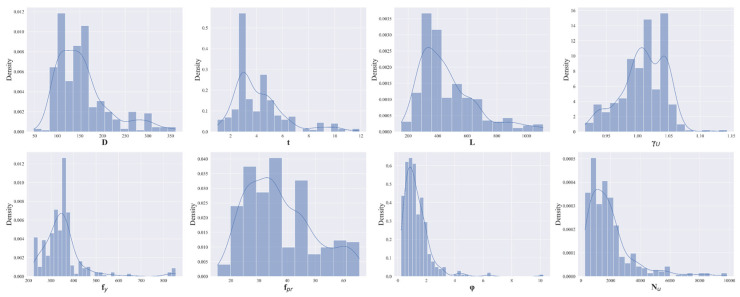
Statistical distributions of input and output features.

Table 1 summarizes the descriptive statistics of the dataset parameters, including the mean, standard deviation, extrema (minimum and maximum), median, and quartiles. The statistical results indicate a wide distribution of feature values, effectively covering the typical parameter ranges encountered in practical CFST engineering applications. Notably, the target variable Nu exhibits a mean of 1917.44 kN, with a variation ranging from 273 kN to 9835 kN. This significant span demonstrates the high representativeness of the dataset, which encompasses diverse samples ranging from small-scale laboratory specimens to large-scale structural members. The frequency distributions of these parameters are illustrated in Figure 1.

### 2.3. Feature Correlation Analysis

**Figure 2:**
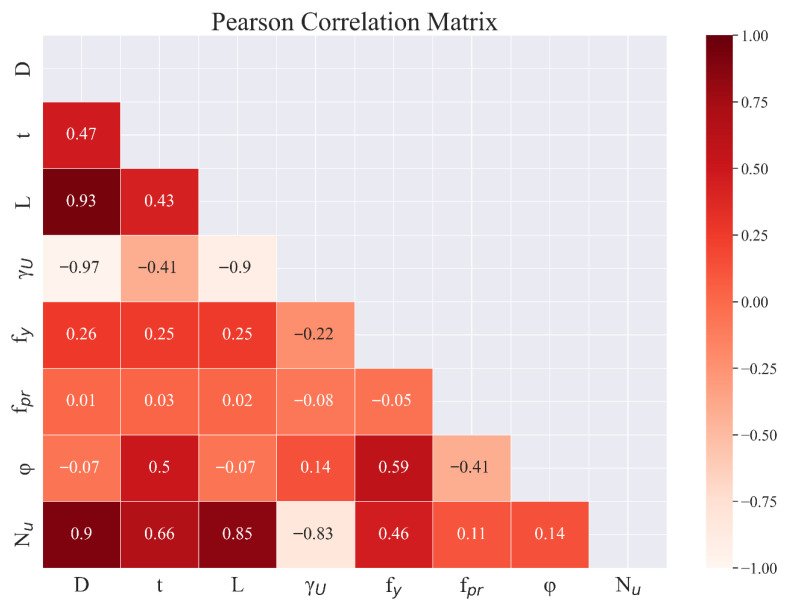
Pearson correlation coefficient matrix.

To elucidate the linear dependencies between the input features and the ultimate axial capacity (*N_u_*), a Pearson correlation analysis was conducted, with the resulting matrix presented in Figure 2. The analysis reveals that the *D* exhibits the strongest positive correlation with *N_u_* (*r* = 0.90), followed closely by the *L* (*r* = 0.85). Notably, a significant collinearity exists between *D* and *L* (*r* = 0.93). This observation reflects engineering practice, where larger diameters are typically associated with longer members to satisfy code-mandated slenderness limits. Furthermore, moderate correlations are observed for t (*r* = 0.66) and *f_y_* (*r* = 0.46). Overall, *N_u_* demonstrates a predominant linear sensitivity to the geometric and material properties of the steel tube, underscoring their critical role in determining axial compressive capacity.

## 3. Theoretical Background of Machine Learning Algorithms

In this study, six representative machine learning algorithms were employed to predict the ultimate axial bearing capacity (*N_u_*) of CFST: LightGBM, Random Forest (RF), Gradient Boosting Decision Tree (GBDT), K-Nearest Neighbors (KNN), CatBoost, and XGBoost. This selection encompasses both tree-based ensemble learning techniques and distance-based instance learning methods, thereby ensuring the capability to accommodate diverse data distributions and capture complex feature dependencies.

### 3.1. LightGBM Model

LightGBM (Light Gradient Boosting Machine) is an efficient machine-learning framework built upon gradient-boosted decision trees. Distinguished by its computational speed and low memory usage, LightGBM implements a histogram-based algorithm that discretizes continuous floating-point feature values into discrete bins. Furthermore, it employs Exclusive Feature Bundling (EFB) to reduce feature dimensionality by bundling mutually exclusive features. Unlike the conventional level-wise growth strategy, LightGBM adopts a leaf-wise tree growth strategy with depth limitations, which typically achieves better accuracy and faster convergence by minimizing the training loss more aggressively. Additionally, Gradient-based One-Side Sampling (GOSS) is incorporated to prioritize training instances with larger gradients, thereby accelerating the learning process while maintaining accuracy. The objective function of the LightGBM model can be formulated as follows:

(1) $${\hat{y}}_{i}=\sum_{k=1}^{K}f_{k}\left(x_{i}\right),\;f_{k}\in F$$

Here, ${\hat{y}}_{i}$ denotes the predicted value, $f_{k}$ represents the *k*-th regression tree, and F is the space of all possible trees. The training process involves minimizing the following objective function:

(2) $$L=\sum_{i=1}^{n}l\left(y_{i},\hat{y}i\right)+\sum k=1^{K}\Omega \left(f_{k}\right)$$

where *l* is the loss function, and Ω serves as the regularization term to prevent overfitting.

### 3.2. Random Forest (RF) Model

Random Forest (RF) is a robust ensemble learning algorithm that enhances generalization by integrating a multitude of independent decision trees through majority voting or averaging. Central to this framework is Bootstrap Aggregation (Bagging), which generates diverse training subsets via sampling with replacement. This is coupled with a random feature selection strategy at each split node to maximize the decorrelation among constituent base estimators. Notably, RF is insensitive to variations in feature scales, rendering data normalization or standardization unnecessary. Additionally, the algorithm offers an intrinsic capability to quantify feature importance by assessing the performance degradation resulting from feature permutation. The mathematical formulation of the RF model is defined as:

(3) $$\hat{y}=\frac{1}{B}\sum_{b=1}^{B}f_{b}\left(x\right)$$

where $\hat{y}$ is the model prediction, $f_{b}$ denotes the predictive function of the *b*-th base learner, and B represents the total number of trees in the random forest.

### 3.3. Gradient Boosting (GB) Model

Gradient Boosting (GB) is an ensemble learning method based on the forward stagewise additive modeling framework. The algorithm iteratively improves predictive performance by fitting new base learners to the negative gradients of the loss function with respect to the current ensemble predictions, thereby correcting the errors made in previous iterations. This training process can be interpreted as performing gradient descent optimization in the function space to minimize a specified loss function. Unlike parallel ensemble strategies like Random Forest, GB adopts a serial iterative approach, forcing subsequent base learners to focus on correcting samples that the current model has not fully fitted, which significantly improves the overall prediction accuracy. Due to its compatibility with arbitrary differentiable loss functions, the GB model demonstrates excellent generalization performance and robustness in both regression and classification tasks. The mathematical expression of the GB is expressed as:

(4) $$F_{m}\left(x\right)=F_{m-1}\left(x\right)+\gamma_{m}h_{m}\left(x\right)$$

Here, $F_{m}\left(x\right)$ denotes the ensemble model after the *m*-th iteration, $h_{m}\left(x\right)$ represents the newly added base learner, and $\gamma_{m}$ is the learning rate controlling the step size of the update. The base learner $h_{m}\left(x\right)$ is trained to approximate the negative gradient of the loss function by minimizing the following objective:

(5) $$h_{m}=\arg\;\min_{h}\sum_{i=1}^{n}{\left[-\frac{\partial L\left(y_{i},F_{m-1}\left(x_{i}\right)\right)}{\partial F_{m-1}\left(x_{i}\right)}-h\left(x_{i}\right)\right]}^{2}$$

### 3.4. K-Nearest Neighbors (KNN) Model

The k-Nearest Neighbors (k-NN) algorithm stands as a quintessential instance-based, non-parametric learning paradigm. Fundamentally, the algorithm operates by identifying the top-k training samples closest to a specific query instance based on a predefined distance metric, subsequently deriving predictions from the target values of these neighbors. In the context of regression tasks, the output is typically determined by computing the average of the target values associated with the k nearest neighbors. Characterized as a “lazy learning” strategy, k-NN eschews an explicit training phase; instead, it retains the entire training dataset and defers computation until the inference stage, utilizing local neighborhood information. This mechanism empowers k-NN to effectively model complex nonlinear mapping relationships. However, the model’s performance exhibits high sensitivity to the hyperparameter k. Furthermore, since the inference phase necessitates exhaustive distance calculations between the query sample and the entire training corpus, the computational overhead can become prohibitive when processing large-scale datasets. The mathematical formulation for k-NN regression is expressed as:

(6) $$\hat{y}=\frac{1}{K}\sum_{i\in N_{K}\left(x\right)}y_{i}$$

where $N_{K}(x)$ denotes the set of indices of the *k* training samples nearest to the query sample x, and $y_{i}$ represents the target value of the *i*-th nearest neighbor.

### 3.5. CatBoost Model

CatBoost is a gradient boosting decision tree algorithm specifically engineered to handle categorical features with high efficiency. While sharing a similar mathematical foundation with standard gradient boosting frameworks, CatBoost distinguishes itself through several innovative mechanisms. It employs symmetric decision trees to mitigate overfitting and utilizes Ordered Boosting combined with Target Statistics to process categorical variables directly. This approach effectively replaces traditional One-Hot Encoding with a more efficient representation. Furthermore, by introducing unbiased gradient estimation, the algorithm addresses the issue of prediction shift, ensuring robust performance even on datasets characterized by complex categorical dependencies. For a categorical feature, CatBoost computes the statistic using the following formula:

(7) $$x_{k,j}=\frac{\sum_{i=1}^{n}\left[y_{i}\cdot \left(x_{i,j}=x_{k,j}\right)\right]+a\cdot b}{\sum_{i=1}^{n}\left(x_{i,j}=x_{k,j}\right)+a}$$

where $x_{i,j}$ denotes the encoded value of the *j*-th feature of the *k*-th sample, and a and b are smoothing parameters.

### 3.6. XGBoost Model

XGBoost (eXtreme Gradient Boosting) is an efficient and scalable distributed gradient boosting system designed for optimized speed and performance. By incorporating L1 and L2 regularization terms into the objective function, the algorithm enhances traditional boosting frameworks, effectively controlling model complexity and suppressing overfitting. XGBoost leverages the second-order Taylor expansion of the loss function to accelerate convergence and adopts a parallel learning scheme based on block structures, combined with pre-sorting and block compression techniques to significantly improve computational efficiency. Furthermore, its built-in sparsity-aware mechanism automatically learns the optimal default branching direction for missing values. The objective function of XGBoost is formally expressed as:

(8) $$L=\sum_{i=1}^{n}l\left(y_{i},{\hat{y}}_{i}\right)+\sum k=1^{K}\left[\gamma T_{k}+\frac{1}{2}\lambda {\left|\left|\omega_{k}\right|\right|}^{2}\right]$$

where *l* is the loss function, $T_{k}$ represents the number of leaf nodes in the *k*-th tree, and γ are regularization parameters, and *λ* denotes the leaf weights.

## 4. Model Optimization Strategies and Evaluation

### 4.1. Input Feature Standardization

Performing Z-Score standardization on the dataset prior to machine learning model training is a critical step for enhancing model robustness and training efficiency. This method effectively eliminates dimensional discrepancies between features by transforming feature values into a standard normal distribution with a mean of 0 and a standard deviation of 1. Its primary functions are as follows:

(1) Mitigating Numerical Instability. Z-Score standardization effectively averts the imbalance in model parameter weight distribution resulting from significant disparities in feature scales. By preventing features with large magnitudes from dominating weight updates during gradient descent, it preserves numerical stability in computations.
(2) Enhancing Convergence Efficiency. By applying an approximately uniform scaling to the feature space, this method ensures that parameter dimensions exhibit comparable scales of variation during optimization, facilitating coordinated gradient updates across all directions. Consequently, the contours within the parameter search space approximate a spherical shape, which significantly improves the convergence efficiency of gradient-based optimization algorithms and accelerates model training.
(3) Strengthening Feature Representation Capabilities. For learning algorithms reliant on distance metrics or inner-product structures, Z-Score standardization bolsters the model’s capacity for comprehensive multi-dimensional feature representation. By eliminating the “pseudo-importance” arising from variations in units and value ranges, it ensures that all dimensions contribute balanced weights to distance calculations, thereby more accurately characterizing the intrinsic similarity structure among samples.
(4) Correcting Regularization Bias. Standardization eliminates model performance biases caused by feature scale heterogeneity. In linear models incorporating regularization terms, the absence of standardization often leads to features with larger scales being disproportionately penalized. Z-Score standardization prevents this systematic bias in parameter estimation, ensuring that regularization constraints are applied equitably across all dimensions.

Extensive empirical studies indicate that, while preserving the relative distributional structure of the original data, Z-Score standardization effectively enhances model generalization performance and training stability on complex, high-dimensional datasets. Its mathematical formulation is expressed as follows:

(9) $$x^{\prime }=\frac{x-\mu }{\sigma }$$

where μ denotes the mean of the dataset *x*, and σ represents the standard deviation of the dataset elements. The term $x^{\prime }$ corresponds to the standardized result, which approximates a standard normal distribution, specifically the $x^{\prime }∼N(0,1)$ distribution.

### 4.2. Hyperparameter Optimization Based on Random Search

The performance of machine learning models relies heavily on the selection of hyperparameters, as appropriate hyperparameters can significantly enhance a model’s predictive accuracy and generalization capability. In this study, hyperparameter optimization is conducted using a five-fold cross-validation (*K* = 5) approach combined with a Random Search algorithm.

#### 4.2.1. Five-Fold Cross-Validation

**Figure 3:**
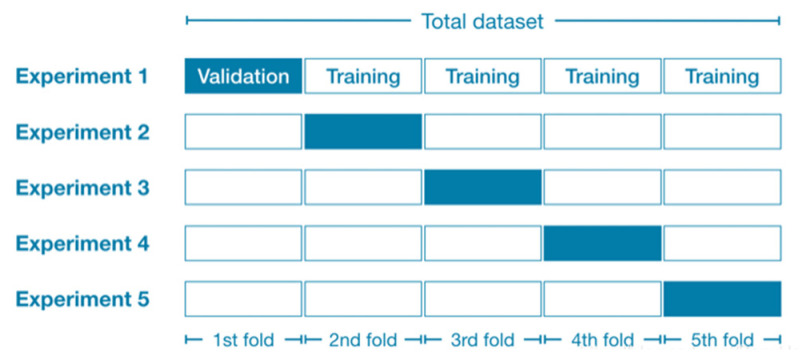
Schematic diagram of five-fold cross-validation.

As illustrated in Figure 3, five-fold cross-validation divides the training dataset into five subsets of approximately equal size. In each iteration, four subsets serve as the training data, while the remaining single subset functions as the validation data. By repeating this process with five different partition combinations, limited data resources are fully utilized, yielding a reliable estimate of model performance. Five-fold cross-validation effectively maximizes the utility of limited training data, prevents overfitting, and significantly reduces random sampling bias, thereby ensuring the stability of the evaluation results. Furthermore, through multiple rounds of validation, it provides a more robust estimate of model performance, offering more accurate evaluation metrics for hyperparameter optimization.

#### 4.2.2. Random Search Algorithm

In this paper, we adopt Random Search over the traditional Grid Search method for hyperparameter optimization. Random Search operates by randomly sampling multiple sets of parameter combinations from a predefined hyperparameter space, subsequently selecting the combination that yields the best performance as the final model configuration. Compared to Grid Search, Random Search demonstrates higher computational efficiency, particularly in high-dimensional parameter spaces. Furthermore, it allows for the exploration of a broader range of parameter combinations, often identifying solutions that are comparable to or better than those found via Grid Search. In this study, the number of iterations for Random Search was set to n_iter = 100 which means that 100 sets of parameter combinations were randomly sampled and evaluated within each model’s hyperparameter space. The calculation formula for Random Search is as follows:

(10) $$\theta^{*}=\arg\times \min_{\theta \sim P(\Theta )}\frac{1}{K}\sum_{k=1}^{K}L\left(D_{k}^{val},A\left(D_{k}^{train},\theta \right)\right)$$

where θ denotes the hyperparameter combination, P(Θ) represents the distribution over the hyperparameter space, $D_{k}^{train}$ and $D_{k}^{val}$ are the training data and validation data for the *k*-th fold, respectively, *A* is the learning algorithm, and *L* denotes the loss function.

Distinct hyperparameter search spaces were established for the six machine learning algorithms [27,28], as detailed in Table 2. These spaces are designed to cover the key tunable parameters of each model, thereby ensuring a comprehensive exploration scope for the Random Search process.

### 4.3. Evaluation Metrics for Machine Learning Models

To comprehensively evaluate model performance, this study utilizes four evaluation metrics: Root Mean Square Error (RMSE), Mean Absolute Percentage Error (MAPE), Mean Absolute Error (MAE), and the Coefficient of Determination (R^2^). These metrics reflect the accuracy and reliability of model predictions from different perspectives. Specifically, RMSE measures the average deviation between predicted and actual values and is more sensitive to large errors. MAPE captures the ratio of prediction error to the actual value, making it suitable for evaluating performance across data of different scales. MAE measures the average absolute difference between predicted and actual values and is less sensitive to outliers. Finally, R^2^ measures the proportion of variance in the target variable explained by the model. The specific calculation formulas are as follows:

(11) $$RMSE\;(\mathrm{MPa})=\sqrt{\frac{1}{n}\times \sum_{i=1}^{n}{\left(y_{i}^{p}-y_{i}\right)}^{2}}$$

(12) $$MAPE\;(\%)=\frac{1}{n}\times \sum_{i=1}^{n}\left|\frac{y_{i}^{p}-y_{i}}{y_{i}}\right|\times 100$$

(13) $$MAE\;(\mathrm{MPa})=\frac{1}{n}\times \sum_{i=1}^{n}\left|y_{i}^{p}-y_{i}\right|$$

(14) $$R^{2}=1-\frac{RSS}{TSS}=1-\frac{\sum_{i=1}^{n}{\left(y_{i}-y_{i}^{p}\right)}^{2}}{\sum_{i=1}^{n}{\left(y_{i}-\bar{y}\right)}^{2}}$$

## 5. Results and Analysis

### 5.1. Basic Experimental Results

To verify the performance of the proposed machine learning models in predicting the ultimate bearing capacity of CFST, basic experiments were conducted on six classic algorithms: LightGBM, RandomForest, GradientBoosting, KNN, CatBoost, and XGBoost. The experimental results are presented in Table 3.

As shown in Table 3, the XGBoost model achieved the best performance on the training set (RMSE = 20.68 kN, MAPE = 0.01, MAE = 8.45 kN and an R^2^ = 1.00). Similarly, the CatBoost and Gradient Boosting models reached an R^2^ of 1.00 on the training set, indicating that these three tree-based ensemble models possess an exceptional fitting capacity for the training data. However, a comparison with the test set reveals a performance gap; for instance, the RMSE of XGBoost increased from 20.68 kN on the training set to 258.66 kN on the test set, while CatBoost increased from 34.45 kN to 223.21 kN. This suggests that under default hyperparameters, the models exhibit a degree of overfitting. This phenomenon is common in tree-based models and stems from high model complexity and insufficient regularization under default settings, leading the models to ‘memorize’ noise in the training data rather than learning the underlying mechanical laws. Additionally, the KNN model performed poorly on both sets, with an RMSE of 486.71 kN on the test set, indicating its weak adaptability to the predictive task in this study.

**Figure 4:**
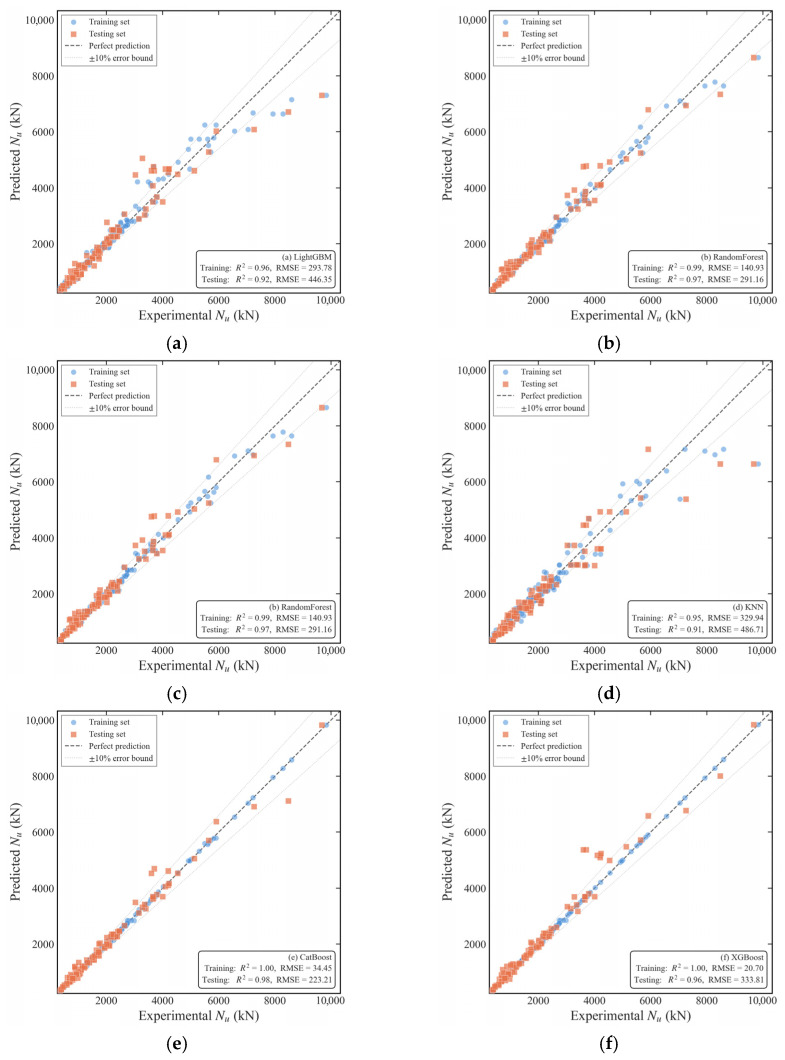
Prediction scatter plots of the basic experiments for six models ((**a**) LightGBM, (**b**) RF, (**c**) GB, (**d**) KNN, (**e**) CatBoost, (**f**) XGBoost).

Figure 4 displays the scatter plots of the predictions from the basic experiments. It is evident from the plots that the GB, CatBoost, and XGBoost models fit the experimental data well, as the prediction points are concentrated near the diagonal line. This demonstrates the robust ability of these three models to predict the ultimate bearing capacity of CFST members.

### 5.2. Results of 5-Fold Cross-Validation and Hyperparameter Optimization

To further improve model performance, this study combined 5-fold cross-validation with random search optimization to identify the optimal hyperparameters. For each machine learning model, a specific hyperparameter space was defined, and the optimal parameter combination within that space was sought using the random search method.

After random search optimization, the optimal parameter combination for the six ML models is shown in Table 4.

### 5.3. Comparison Between Optimized and Original Models

Table 5 presents the performance comparison between the optimized model and the original model on the test set. It illustrates that the performance of all models on the test set improved after hyperparameter optimization, and the overfitting observed in the baseline experiments was significantly mitigated. The R-CatBoost model demonstrated the superior performance, with the RMSE decreasing to 174.29 kN and the R^2^ increasing to 0.99, representing the highest predictive accuracy and generalization capability. The most significant improvement was observed in the R-LightGBM model, where the RMSE dropped from 446.35 kN to 272.16 kN, a reduction of 39.0%. This demonstrates that the optimization strategy—utilizing Random Search and 5-fold cross-validation—effectively controlled model complexity and achieved an optimal balance between fitting capacity and generalization performance. This confirms the critical role of hyperparameter optimization in mitigating overfitting. While the R-KNN model showed improvement, it remained the weakest performer, suggesting that the KNN algorithm may not be well-suited for the specific data features of this study.

**Figure 5:**
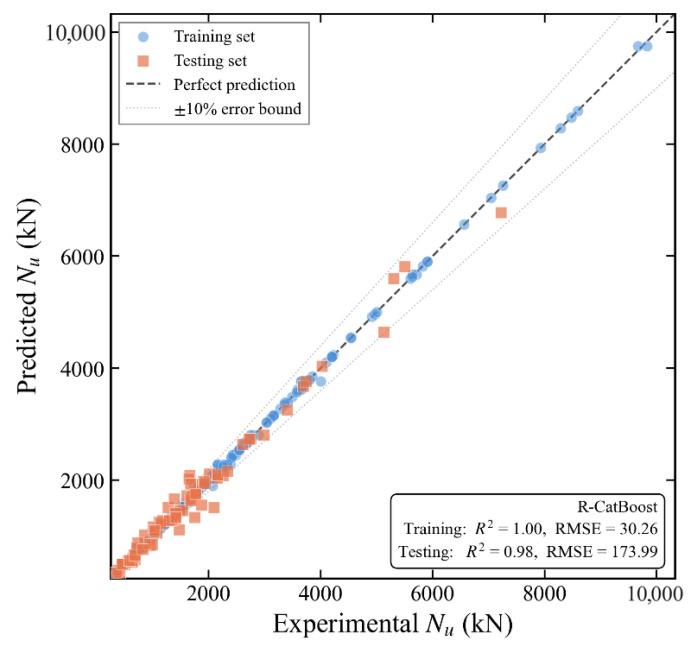
Actual vs. Predicted Values for the Improved Models.

Figure 5 illustrates the relationship between the actual and predicted values for all improved models. As observed from the figure, the prediction points of the R-CatBoost model are closest to the diagonal line and exhibit the most concentrated distribution, which further demonstrates its superior predictive performance.

### 5.4. Comparison of This Study with Existing Literature

Based on the comparative analysis of the research findings in Table 6, the following conclusions can be drawn across three key dimensions:

(1) Data-Driven Methodology: This study relies exclusively on 438 authentic experimental cases, prioritizing the physical reliability and empirical groundedness of the data. In contrast, Kazemi [19] and Xie [31] extensively utilized Finite Element Analysis (FEA) simulation data to compensate for the scarcity of experimental records. Kazemi [19] further advanced this by incorporating synthetic data generation techniques (GANs/VAEs) to address the challenges of small-sample learning.
(2) Algorithmic Evolution: There is a clear transition in the complexity of the predictive models used. Early research, such as Lyu [30], focused on the heuristic optimization of individual base models (e.g., SVR). However, more recent studies—including this work, Xie [31], and Kazemi [19]—have shifted toward Ensemble Learning frameworks. Algorithms like CatBoost and XGBoost leverage multi-model synergy to significantly enhance generalization accuracy and predictive robustness.
(3) Validation Depth: This study distinguishes itself by utilizing SHAP interpretability analysis to demonstrate the scientific alignment between machine learning results and classical structural mechanics, moving beyond simple curve fitting. For comparison, Li [29] emphasized the applicability of models to large-scale external specimens, while Zhao [32] focused on assessing model accuracy through in situ experimental validation.

## 6. SHAP-Based Interpretability Analysis

SHAP (SHapley Additive exPlanations) is a unified interpretation framework based on cooperative game theory, which is capable of measuring the marginal contribution of each feature in a single prediction. Given that the R-CatBoost model demonstrated the optimal performance among all models, this study employs SHAP values to interpret the R-CatBoost model. Specifically, the influence of each input feature on the prediction of the ultimate bearing capacity of CFST is systematically analyzed from both global and local perspectives.

### 6.1. SHAP Summary Plot

**Figure 6:**
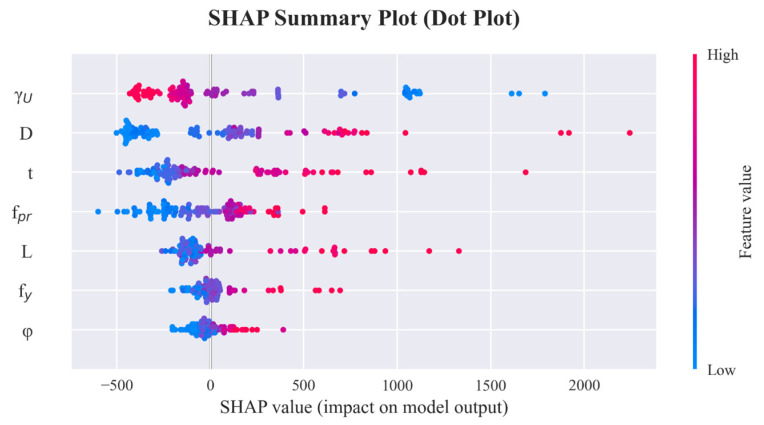
SHAP Summary Plot.

The SHAP summary plot is illustrated in Figure 6. The D and t exhibit the widest range of SHAP value distribution. Specifically, scatter points corresponding to high feature values are predominantly located in the positive SHAP region, whereas those for low values are concentrated in the negative region. This indicates that larger diameters and wall thicknesses significantly enhance the predicted ultimate bearing capacity, while smaller dimensions tend to reduce the predicted values. These findings suggest that geometric dimensions are the dominant factors controlling the ultimate bearing capacity of CFST members. Overall, these observations are consistent with empirical engineering knowledge, identifying section size and material strength as decisive factors, whereas member length and size effects play a secondary role.

### 6.2. SHAP Feature Importance Plot

**Figure 7:**
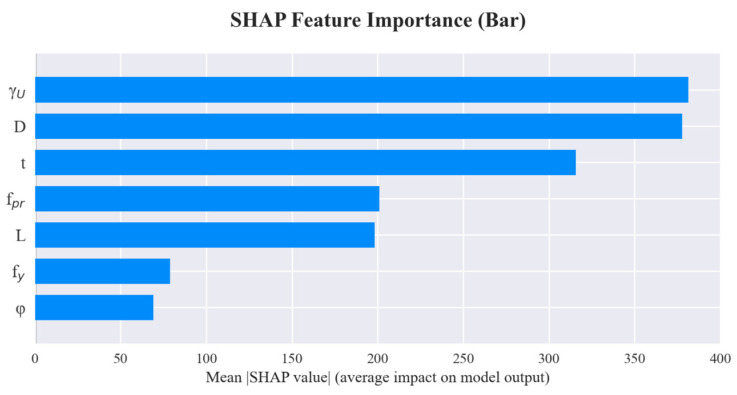
SHAP Feature Importance Plot.

Consistent with the results from the summary plot, Figure 7 indicates that *D* and *t* are the two most important features influencing model predictions, with the sum of their absolute SHAP values being significantly higher than that of other features. This reconfirms the decisive impact of steel pipe diameter and wall thickness on the ultimate bearing capacity.

### 6.3. SHAP Dependence Plot

**Figure 8:**
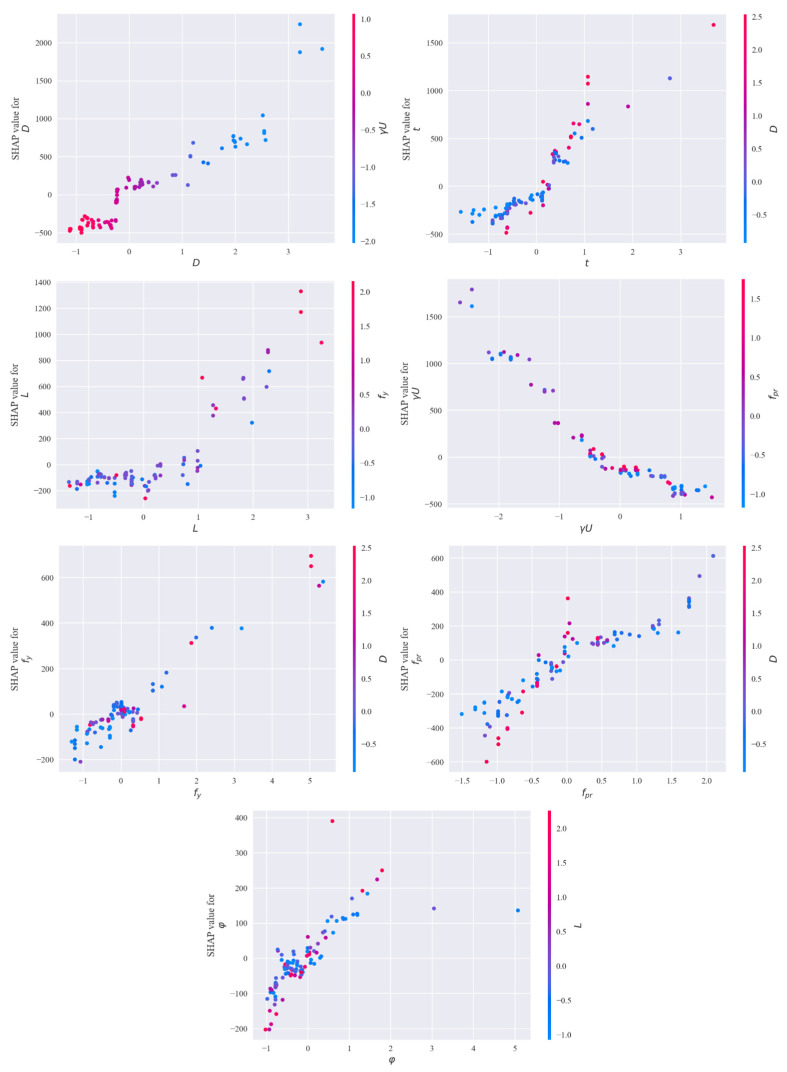
SHAP Dependence Plot.

To intuitively analyze the impact of feature value variations on prediction results and feature interactions, SHAP dependence plots for key features were generated, as shown in Figure 8. The results indicate the following:

(1) *D* and *t*: Their SHAP values generally show an upward trend as the feature values increase, indicating that larger diameters and thicker walls typically enhance the ultimate bearing capacity.
(2) *f_y_*: This feature exhibits a non-linear relationship with SHAP values. The marginal influence is more sensitive in the medium strength range, while it plateaus at lower or higher ranges. This suggests that capacity gains in these extreme ranges may be limited by other failure modes or concrete/construction constraints.
(3) *f_pr_*: There is an overall positive correlation, yet with fluctuations, reflecting that its effect is influenced by the confinement status and parameter combinations.
(4) *L*: The influence of length is more complex. For small LL, SHAP values fluctuate near zero, showing minimal impact. However, once LL exceeds a certain threshold, SHAP values drop sharply into the negative range. This accurately captures the Euler Buckling phenomenon: as the slenderness ratio increases, the failure mode shifts from material failure to instability failure, and the P−δ effect significantly reduces the ultimate bearing capacity.

While traditional empirical formulas typically describe this behavior using a stability reduction factor (*φ*), the CatBoost model has automatically learned this physical law from the data. Overall, these dependence plots quantitatively reveal the non-linear mechanisms of geometric dimensions, material strength, and slenderness ratio on bearing capacity from a data-driven perspective, providing a reference for the rational selection of section and material parameters. These dependence plots quantitatively reveal the nonlinear mapping relationships between the feature parameters and the predicted bearing capacity, with the predicted trends showing a high degree of correlation with classical mechanical theories. In particular, the model successfully captures the Euler buckling and *P-δ* effects resulting from an increase in the slenderness ratio. This demonstrates that the data-driven model possesses robust physical consistency and engineering reliability.

### 6.4. SHAP Value Heatmap

**Figure 9:**
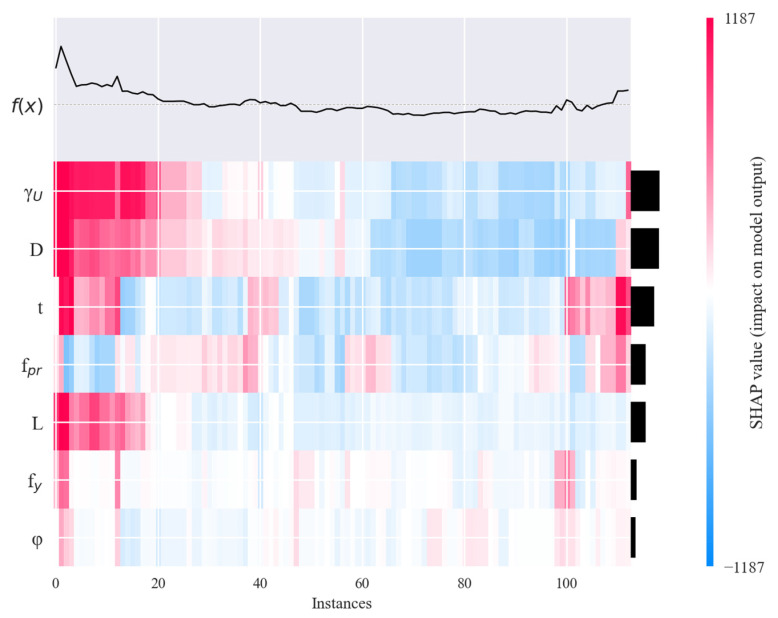
SHAP Value Heatmap.

As shown in Figure 9, features *D* and *t* exert the strongest influence on the prediction results, which is consistent with previous analyses. Furthermore, the heatmap reveals the interaction patterns among different features, further illustrating the combined effect of various feature combinations on the prediction outcome.

## 7. Conclusions

This study addresses the prediction of the ultimate bearing capacity of Concrete-Filled Steel Tubular (CFST) members. Various machine learning models were developed and compared, followed by a systematic interpretability analysis based on the optimal model. The main conclusions are drawn as follows:

(1) Through the training and evaluation of multiple machine learning models, and utilizing random search combined with five-fold cross-validation for hyperparameter optimization, the R-CatBoost model achieved optimal performance on the test set. With an RMSE of 174.29, MAPE of 0.06, MAE of 107.30, and a coefficient of determination (*R*^2^) as high as 0.99, the model demonstrates the ability to predict the ultimate bearing capacity of CFST members with high precision while maintaining strong generalization capabilities on unseen data. Compared with traditional empirical formulas and other mainstream machine learning methods, R-CatBoost exhibits significant advantages in terms of both accuracy and robustness, serving as a reliable numerical tool for engineering design and assessment.
(2) Global interpretation results based on the SHAP framework indicate that the D and t are the primary factors influencing the ultimate bearing capacity, followed by the *f_y_* and the *f_pr_*. The impacts of L and the $\gamma_{U}$ are relatively minor. This ranking of feature importance is highly consistent with existing theories and engineering experience: larger cross-sectional dimensions and thicker steel tube walls significantly enhance the bearing and confinement capacities of the member, while increasing steel and concrete strengths effectively raises the ultimate bearing capacity within a certain range. This consistency demonstrates that the R-CatBoost model not only possesses excellent numerical fitting performance but its internal decision logic also aligns with structural mechanical mechanisms, thereby enhancing the credibility of the model in engineering applications.
(3) Systematic hyperparameter optimization plays a crucial role in enhancing model performance. By combining random search with five-fold cross-validation, this study effectively improved the generalization ability of multiple models. The improvement was particularly notable for the LightGBM model, which saw a 39.0% reduction in RMSE. This indicates that rational hyperparameter settings can fully unleash model potential while preventing overfitting, making it an indispensable step when applying machine learning models to practical engineering problems.

## Figures and Tables

**Table 1:** Summary statistics of the database parameters.

Features	*D*/mm	*t*/mm	*L*/mm	*γ_U_*	*f_y_*/MPa	*f_pr_*/MPa	*Φ*	*N_u_*/kN
Max	361	11.88	1113	1.14	853	65.60	10.21	9835
Min	48	1.00	153	0.92	223	15.28	0.23	273
Mean	155.77	4.18	464.81	1.01	352.95	37.41	1.30	1917.44
Std. Dev	59.03	1.98	194	0.04	100.48	12.02	0.96	1552.45
Median	140	3.56	419	1.0	347.90	34.72	1.09	1580
25%	109.66	2.97	312	0.99	303.50	27.02	0.71	870.50
75%	172.80	5.01	572.10	1.04	369.50	44.46	1.67	2270.25

**Table 2:** Hyperparameter spaces for the machine learning algorithms.

Algorithm	Hyperparameter	Search Space
Light GBM	colsample_bytree	[0.6–1.0]
	learning_rate	[0.01–0.3]
	max_depth	[3–15]
	min_child_samples	[1–50]
	n_estimators	[50–500]
	num_leaves	[20–1500]
	reg_alpha	[0–2]
	reg_lambda	[0–2]
	subsample	[0.5–1.0]
Random Forest	bootstrap	[True, False]
	max_depth	[3–15]
	max_features	[0.1–1.0]
	min_samples_leaf	[1–10]
	min_samples_split	[2–20]
	n_estimators	[50–500]
GradientBoosting	learning_rate	[0.01–0.3]
	max_depth	[3–10]
	max_features	[0.1–1.0]
	min_samples_leaf	[1–20]
	min_samples_split	[2–20]
	n_estimators	[50–500]
	c	[0.5–1.0]
KNN	leaf_size	[5–100]
	n_neighbors	[1–20]
	p	[1, 2]
CatBoost	n_estimators	[100–2000]
	learning_rate	[0.01–0.3]
	l2_leaf_reg	[1–10]
	depth	[3–10]
	border_count	[32–255]
	bagging_temperature	[0–10]
XGBoost	colsample_bytree	[0.3–1.0]
	gamma	[0–1]
	learning_rate	[0.01–0.3]
	max_depth	[3–10]
	n_estimators	[50–1000]
	reg_alpha	[0–2]
	reg_lambda	[0–2]
	subsample	[0.5–1.0]

**Table 3:** Simulation results on the training and validation sets.

Model	Evaluation Indicators				
	Dataset	RMSE	MAPE	MAE	R^2^
LightGBM	Training	293.78	0.06	126.92	0.96
	Validation	446.35	0.10	228.22	0.92
RandomForest	Training	140.21	0.03	68.40	0.99
	Validation	290.27	0.09	173.81	0.97
GradientBoosting	Training	66.49	0.04	51.34	1.00
	Validation	216.23	0.09	136.10	0.98
KNN	Training	329.94	0.08	165.13	0.95
	Validation	486.71	0.12	262.47	0.91
CatBoost	Training	34.45	0.02	25.49	1.00
	Validation	223.21	0.06	112.43	0.98
XGBoost	Training	20.68	0.01	8.45	1.00
	Validation	258.66	0.07	132.62	0.97

**Table 4:** The Optimal Parameter Combination for the six ML models.

Algorithm	Hyperparameter	Optimal Parameters
Light GBM	colsample_bytree	0.87
	max_depth	13
	n_estimators	238
	reg_alpha	1.56
	subsample	0.56
	learning_rate	0.29
	min_child_samples	12
	num_leaves	1075
	reg_lambda	1.56
Random Forest	bootstrap	FALSE
	max_features	0.79
	min_samples_split	2
	max_depth	11
	min_samples_leaf	1
	n_estimators	288
GradientBoosting	learning_rate	0.17
	max_features	0.68
	min_samples_split	16
	subsample	0.96
	max_depth	5
	min_samples_leaf	11
	n_estimators	298
KNN	leaf_size	54
	n_neighbors	1
	p	1
CatBoost	n_estimators	2000
	l2_leaf_reg	3
	border_count	128
	learning_rate	0.3
	depth	4
	bagging_temperature	1.5
XGBoost	subsample	0.6
	reg_alpha	0.1
	max_depth	3
	gamma	0.5
	reg_lambda	0.1
	n_estimators	500
	learning_rate	0.1
	colsample_bytree	1

**Table 5:** Performance Comparison Between the Original and Improved Models on the Test Set.

Model	Evaluation Indicators			
	RMSE	MAPE	MAE	R^2^
LightGBM	446.35	0.10	228.22	0.92
R-LightGBM	272.16	0.09	157.08	0.97
RandomFores	290.27	0.09	173.81	0.97
R-RandomFores	280.57	0.07	140.32	0.97
GradientBoosting	216.23	0.09	136.10	0.98
R-GradientBoosting	223.63	0.07	127.61	0.98
KNN	486.71	0.12	262.47	0.91
R-KNN	419.83	0.08	184.50	0.93
CatBoost	223.21	0.06	112.43	0.98
R- CatBoost	174.29	0.06	107.30	0.99
XGBoost	258.66	0.07	132.62	0.97
R- XGBoost	218.35	0.08	130.54	0.98

**Table 6:** Comparison of the Results of This Study with Relevant Recent Research Findings.

Reference	Machine Learning Methods	Dataset Source & Scale	Validation & Evaluation Methods
This Study	R-CatBoost, LightGBM, RF, GB, KNN, XGBoost	38 independent test programs; 438 data points	Train/Test = 70/30; 5-fold CV; SHAP interpretability analysis
Kazemi [19]	Ensemble learning framework (BR, XGBoost, GBM, RF, etc.)	88 numerical models/12 tests; 2000 data points	Train/Test = 80/20; Multi-dimensional metrics; GUI interface showcase
Li [29]	PSO-GPR, BPNN, SVR, GPR	15 independent test programs; 162 data points	Experimental data of large-scale members
Lyu [30]	SCA-SVR, ANN, RF, MLR	Experimental data; 478 data points	Train/Test = 70/30; 100 random trials for stability; Inverse design parameter prediction
Xie [31]	XGBoost, RF, LightGBM, AdaBoost, CatBoost, LSTM	66 tests/134 numerical simulations; 200 data points	Train/Test = 70/30; Taylor diagram comparison; SHAP interpretability analysis
Zhao [32]	(Multilevel Extension) + AHP	25 independent tests; 449 data points	3-specimen uniaxial compression test

## Data Availability

The original contributions presented in this study are included in the article. Further inquiries can be directed to the corresponding author.

## Appendix A

As detailed in Table A1, the original sources for the 438 datasets (previously cited collectively in Ref. [26]) have been explicitly identified. The compiled database encompasses a wide range of geometric and material properties: the outer diameter (*D*) ranges from 48 mm to 361 mm, the wall thickness (*t*) from 1 mm to 11.88 mm, and the member length (*L*) from 153 mm to 1113 mm. Furthermore, the concrete grades span from C20 to C60. These parameters effectively cover the majority of standard CFST configurations commonly employed in structural engineering practice.

**Table A1:** Source of the Original Dataset and Characteristics of Data Distribution.

No.	References	D/mm	t/mm	L/mm	Sample Size	Percentage/%
1	Wang [33]	133.1~167.8	3.28~5.44	396~504	38	8.68
2	Lam D [34]	114~115	3.75~5.02	300	5	1.14
3	Ho J C M [35]	114~69.2	2.86~9.96	248~420	12	2.74
4	Schneider S P [36]	140	3~6.68	467~472	3	0.68
5	Miao [37]	100~273	1.45~8.75	309~1113	45	10.27
6	Tang [38]	92.0~210.0	1.5~4.0	276.0~630.0	16	3.65
7	Cai [39]	300/166	3/5	1000/660	4	0.91
8	Ding [40]	165~219	2.72~4.78	500~650	13	2.97
9	Gu [41]	152~166	4.5~10	480~500	6	1.37
10	Jiang [42]	166~202	3.0~4.5	500~600	12	2.74
11	Yao [43]	100~200	3	300~600	4	0.91
12	Zhao [44]	90	1.2~1.5	270	9	2.05
13	Tan [45]	133	4.7	465	4	0.91
14	Motoi [46]	101.6~139.8	2.37~2.99	305~419	18	4.11
15	He [47]	150~273	3.0~4.5	465~704	21	4.79
16	Chen [48]	100	1.6~2.5	300	8	1.83
17	Goode [49]	48~165	2.38~6.2	192~660	24	5.48
18	Sakino [50]	174~179	3.0~9.0	360	8	1.83
19	Luksha [51]	159~1020	5.07~13.25	477~3060	18	4.11
20	Sakino [52]	101.8	2.94~5.7	200	32	7.31
21	Huang [53]	159~164	3.8~6.3	520	12	2.74
22	Shea O [54]	165/190	1.94/2.82	580.5/663.5	6	1.37
24	Gardner N J [55]	76.5~152.6	1.7~4.09	153~305	8	1.83
25	Kato [56]	297.0~301.5	4.5~11.88	891~905	9	2.05
26	Yamamoto [57]	101.3~318.5	3.02~10.38	304~956	13	2.97
27	Weng [58]	200/280	5/4	600/840	4	0.91
28	Johansson [59]	159	5~10	650	3	0.68
29	Gu [60]	127~133	1.5~4.5	400	4	0.91
30	Yu [61]	100/200	3	300/600	16	3.65
31	Gupta [62]	89.3/112.6	2.89/2.74	340	16	3.65
32	Abed [63]	114~167	3.1~5.6	228~334	6	1.37
33	Liao [64]	180	3.8	720	2	0.46
34	Zhao [44]	140.0~216.3	4.5~8.2	420.0~648.9	24	5.48
35	Ye [65]	165	2.37	615	2	0.46
36	Ekmekyapar [66]	114.3	2.74/5.9	300	4	0.91
37	Chang [67]	111.64/113.64	1.9/3.64	400	6	1.37
38	Han [68]	120	2.65	360	2	0.46
39	Uenaka [69]	157.7	2.14	450	1	0.23
Total					438	100
